# Emerging mycotoxin occurrence in chicken feed and eggs from Algeria

**DOI:** 10.1007/s12550-024-00537-2

**Published:** 2024-05-16

**Authors:** Chahinez Laouni, Francisco J. Lara, Ahmed Messai, Sara Redouane-Salah, Maykel Hernández-Mesa, Laura Gámiz-Gracia, Ana M. García-Campaña

**Affiliations:** 1 DEDSPAZA Laboratory, Department of Agronomical Sciences, Faculty of Exact Sciences and Natural and Life Sciences, University of Biskra, Biskra, Algeria; 2 Department of Analytical Chemistry, Faculty of Sciences, University of Granada, Granada, Spain; 3 PIARA Laboratory, Department of Agronomical Sciences, Faculty of Exact Sciences and Natural and Life Sciences, University of Biskra, Biskra, Algeria

**Keywords:** Mycotoxins, Poultry feed, Eggs, Enniatins, Beauvericin

## Abstract

Poultry farming has developed into one of Algeria’s most productive industrial farming because of the growing demand for sources of protein among Algerian society. Laying hen feed consists mainly of cereals, which can be contaminated with molds and subsequently with their secondary metabolites known as mycotoxins. These later can pose a serious danger to the production and quality of eggs in the commercial layer industry. This work focuses on the detection of emerging mycotoxins, mainly enniatins (ENNs) and beauvericin (BEA), in poultry feed and eggs from different locations in Algeria. Two different QuEChERS-based extractions were established to extract ENNs and BEA from chicken feed and eggs. The determination of mycotoxin occurrence was achieved by a UHPLC-MS/MS method using 0.1% (v/v) formic acid in water and MeOH as mobile phase, an ESI interface operating in positive mode, and a triple quadrupole mass spectrometer operating in MRM for the detection. Matrix-matched calibration curves were carried out for both matrices, obtaining good linearity (*R*^2^ > 0.99). The method performance was assessed in terms of extraction recovery (from 87 to 107%), matrix effect (from − 47 to − 86%), precision (RSD < 15%), and limits of quantitation (≤ 1.1 µg/kg for feed and ≤ 0.8 µg/kg for eggs). The analysis of 10 chicken feed samples and 35 egg samples composed of a 10-egg pool each showed that ENN B_1_ was the most common mycotoxin (i.e., found in 9 feed samples) with contamination levels ranging from 3.6 to 41.5 µg/kg, while BEA was detected only in one feed sample (12 µg/kg). However, eggs were not found to be contaminated with any mycotoxin at the detection limit levels. Our findings indicate that the searched mycotoxins are present in traces in feed and absent in eggs. This can be explained by the application of a mycotoxin binder. However, this does not put a stop on the conduction of additional research and ultimately setting regulations to prevent the occurrence of emerging mycotoxins.

## Introduction

Fungi are microorganisms that have a wide range of beneficial and harmful effects (Khalifa et al. 2022). In addition to causing reductions in grain yield and quality, some mold species are also responsible for the contamination of grains with a variety of mycotoxins (Balendres et al. 2019; Shar et al. 2020). These secondary metabolites can produce fatal or side effects when ingested or consumed by humans or animals (Ayofemi Olalekan Adeyeye 2020). They can enter the food chain directly through contaminated food or indirectly through contaminated animal products that were fed with infected grains (Decleer et al. 2016). Poultry are very sensitive to mycotoxins (Hassan et al. 2012; Magnoli et al. 2019). Therefore, they may have relatively high mycotoxin levels in their blood during egg production, meaning that there will be a high risk of contamination of the eggs produced (Greco et al. 2014; Pettersson 2012).

Research on the most common mycotoxins, such as ochratoxin A, aflatoxins, and trichothecenes, has greatly risen over the last 10 years as a result of their increasing prevalence throughout the whole food chain (Agriopoulou 2016). However, less research has been done on the occurrence of emerging mycotoxins (Juan et al. 2008; Serrano et al. 2013). *Fusarium* species produce mycotoxins called enniatins (ENNs) and beauvericin (BEA), which have recently attracted researches due to the vast range of biological actions they exhibit (Křížová et al. 2021). Among the 29 known analogues, ENN A, A_1_, B, and B_1_ are the most frequently detected ENNs in cereal grains (Gautier et al. 2020). BEA and ENNs, whose lipophilic qualities may cause their accumulation in some animal tissues, are known to pass from feed to animal-derived foods. Indeed, BEA and ENNs have been found in laying hen eggs, with accumulation of these mycotoxins in the yolk and in various tissues of turkeys and broilers (Jestoi et al. 2009; Křížová et al. 2021).

The European Commission has established maximum limits for five mycotoxins, including aflatoxin B_1_, deoxynivalenol, zearalenone, fumonisin B_1_ + B_2_, and ochratoxin A, when it comes to animal feed (Kebede et al. 2020; Luo et al. 2021; Streit et al. 2012). However, BEA and ENN levels in food and feed products around the world do not have established regulatory maximum limits (Eskola et al. 2020; Leatherhead Food Research Association 2010). Initially, the second metabolites of *Fusarium* such as ENNs, BEA, and other compounds were grouped under the term “emerging mycotoxins” (Jestoi 2008). Later, the “emerging” term refers to mycotoxins that are not subjected to regulation (Christiane Gruber-Dorninger et al. 2017).

One strategy to reduce exposure of animals to mycotoxins is to decrease the bioavailability of mycotoxins by incorporating various detoxifying agents into animal feed. Mycotoxin binders are designed to sequester different mycotoxins (Sabater-Vilar et al. 2007). The role of these agents has been assessed in vitro and in vivo in several studies (Pappas et al. 2016; Tapingkae et al. 2022; Van Rensburg et al. 2006). The results suggested that the presence of various toxins and the structure of the binder may be crucial elements for the best performance of broilers during mycotoxicosis. Thus, in another study, it was determined that the inclusion of commercial toxin binders in feed containing aflatoxin B_1_ minimizes the negative effects of the toxins and may help with the issue of aflatoxicosis in early broiler chicks (Nazarizadeh and Pourreza 2019).

A multitude of studies in Algeria have extensively investigated mycotoxin detection across diverse matrices (Ait Mimoune et al. 2016, 2018; Redouane-Salah et al. 2015). Additionally, research endeavors examining mycotoxin contamination in animal feeds have primarily centered on established toxins like aflatoxins and ochratoxin A (OTA). For example, Tantaoui-Elaraki et al. (2018) investigated aflatoxigenic fungi in feed, while other comprehensive studies across North Africa have focused on quantifying AFB1 and OTA levels, especially in poultry feeds (Kichou and Walser 1993; Benkerroum and Tantaoui-Elaraki 2001; Zinedine et al. 2007; Sifou et al. 2016; Gruber-Dorninger et al. 2018). These works consistently emphasize the prevalence of aflatoxins as major feed contaminants in the region. However, there has been limited research attention on emerging mycotoxins, including enniatins (ENNs) and beauvericin (BEA), despite their known risks. Surprisingly, even though mycological surveys (Benkerroum and Tantaoui-Elaraki 2001) have isolated *Fusarium* strains capable of producing ENNs and BEA from North African maize, a common feed component, no studies have specifically analyzed feeds for these emerging toxins. Determining the presence of emerging mycotoxins would provide a more comprehensive risk profile and augment the existing data on established contaminants like AFB1 and OTA.

This study focuses on the detection of emerging mycotoxins in eggs and poultry feed collected from various farms located in the Eastern part of Algeria. The main motivation behind this study is the fact that Algerians consume a lot of eggs and poultry (Berrama et al. 2021; Kaci 2015), so it is important to determine the likelihood that they are exposed to emerging mycotoxins present in their main source of protein. Further, this study was driven by the fact that none of the researches conducted in Algeria investigated emerging mycotoxins in laying hence feed and eggs.

## Materials and methods

### Reagents and chemicals

All reagents were of analytical reagent grade whereas solvents were LC–MS grade. Formic acid eluent additive for LC–MS, methanol (MeOH), and acetonitrile (MeCN) were obtained from VWR. Formic acid (analysis grade) was supplied by Merck (Darmstadt, Hesse, Germany), and magnesium sulfate anhydrous, potassium chloride, sodium sulfate anhydrous, and sodium acetate anhydrous were supplied by Panreac (Barcelona, Catalonia, Spain). Sodium citrate tribasic dehydrate was acquired from Sigma-Aldrich. C18 and primary secondary amine were supplied by Agilent Technologies (Waldbronn, Baden-Württemberg, Germany).

Individual standards (powder) of ENN A, ENN A_1_, ENN B, ENN B_1_, and BEA were obtained from Sigma-Aldrich. Stock solutions were prepared at 1000 mg/L in MeCN. Intermediate working solutions of a mixture of mycotoxins in MeCN (10 mg/L of ENN A, ENN A_1_, ENN B, ENN B_1_, and BEA) were prepared by combining suitable aliquots of each individual standard stock solution. These solutions were stored at − 20 °C.

Ultrapure water (Milli-Q Plus system, Millipore, Bedford, MA, USA) was used throughout the work. Nylon syringe filters (13 mm, 0.22 µm, VWR) were used for filtration of extracts prior to the injection into the chromatographic system.

### Instrumentation and equipment

UHPLC-MS/MS analyses were performed on an Agilent 1290 Infinity LC (Agilent Technologies, Waldbronn, Baden-Württemberg, Germany), coupled to an API 3200 triple quadrupole mass spectrometer (AB SCIEX, Toronto, Ontario, Canada) with electrospray ionization (ESI). The chromatographic separation was performed using an Agilent Zorbax Eclipse Plus RRHD C18 column (50 × 2.1 mm, 1.8 µm; Agilent Technologies). Analyst software (version 1.6.3, AB Sciex, Darmstadt, Germany) was used for data acquisition and analysis. Data was acquired applying the multiple reaction monitoring mode (MRM), and ESI in positive mode was selected (Arroyo-Manzanares et al. 2019).

**Fig. 1:**
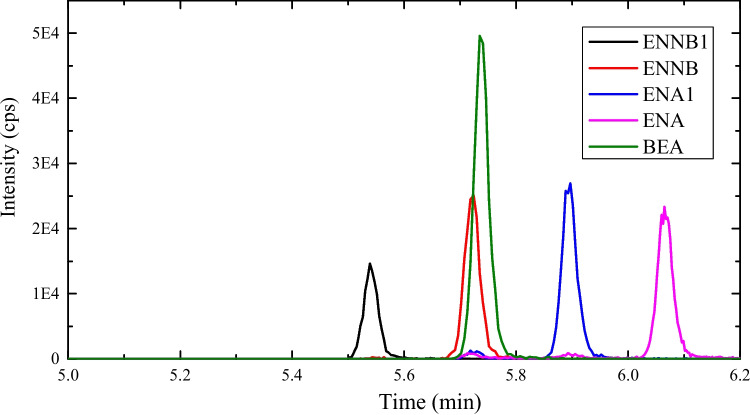
Chromatogram of a standard mixture solution of the five emerging mycotoxins (ENN A, ENN A_1_, ENN B, ENN B_1_, and BEA) at a concentration of 16 µg/L

Sample analyses were carried out by UHPLC-MS/MS using a concentration gradient program and 0.1% formic acid (aqueous) and MeOH as mobile phase. A mobile phase flow rate of 0.4 mL/min was selected. The gradient elution program was established as follows: 0 min, 5% B; 1 min, 50% B; 2 min, 72% B; 4 min, 80% B; and 6 min, 90% B, then back to 5% B in 0.2 min. The temperature of the column was kept at 35° C, and the injection volume was 5 µL. Figure 1 shows a chromatogram of standard mixture of the five emerging mycotoxins (ENN A, ENN A_1_, ENN B, ENN B_1_, and BEA).

In the sample treatment, an evaporator system (System EVA-EC, from VLM GmbH, Bielefeld, Germany), a vortex-2 Genie (Scientific Industries, Bohemia, NY, USA), a universal 320R centrifuge (Hettich ZENtrifugen, Tuttlingen, Germany), and Benchmixer_multitube vortexer (Benchmark) were used. For sample lyophilization, an alpha 1–2 freeze dryer with 316 stainless steel tank, coil with large exchange surface, defrosting (LSCBasic version), was used.

### Sampling and sample preparation

A total of 350 eggs and 10 different chicken feed samples were analyzed. Eggs and chicken feed were randomly collected from different layer farms spread over four states all located in the Eastern part of Algeria. Hence, from each farm, a number of eggs were collected and then 10 eggs were selected and mixed using a blender then a quantity of 50 mL of the mixture was taken to obtain a single sample. Therefore, each sample is from a different farm or from the same farm but from a different egg batch or a different production building. Thus, a total of 10 farms were visited along with eggs collected randomly from the market. Regarding the feed, from each visited farm, feed was collected. Thereafter, each feed sample corresponds to a farm from which eggs were collected. Table 1 presents detailed information on sample collection.

**Table 1:** Origin and distribution of the analyzed samples

State	Source	Number of collected eggs	Number of samples pooled	Number of collected feed samples
Guelma	Farm 1	20	2	1
	Farm 2	40	4	1
	Farm 3	30	3	1
Biskra	Farm 4	30	3	1
	Farm 5	30	3	1
	Farm 6	20	2	1
	Market 1	30	3	/
	Market 2	10	1	/
	Market 3	10	1	/
Batna	Farm 7	30	3	1
	Farm 8	30	3	1
	Farm 9	30	3	1
Oum El Bouaghi	Farm 10	40	4	1
Total	10 farms and 3 markets	350	35	10

The chicken feed samples were kept at 4 °C until sample preparation and analysis. In this sense, the eggs were lyophilized according to the modified method of Tomczyk et al. (2019) and selecting a pressure of 0.005 mBar, and a temperature of − 80 °C. Then, the lyophilized eggs were stored at a temperature of − 20 °C.

For the extraction, two different QuEChERS-based extractions were established to extract ENNs and BEA from chicken feed and eggs, respectively.

### Chicken feed extraction

Feed samples were extracted following the method of Mahdjoubi et al. (2020), with some modifications. Eight milliliters of water was added to 2 g of grounded sample weighed in a polypropylene centrifuge tube (50 mL), and subsequently vortexed for 1 min. After, 10 mL of 5% formic acid in MeCN was added to the mixture, and vortexed for 3 min. Then, 4 g of MgSO_4_, 1 g of NaCl, 1 g of sodium citrate, and 0.5 g of disodium hydrogen citrate sesquihydrate were added and shaken vigorously for 2 min. After centrifugation at 4500 rpm for 5 min, 2 mL of the supernatant layer was transferred to a 4-mL vial, and subsequently evaporated to dryness under a gentle stream of nitrogen. The last step was the reconstitution to a final volume of 1 mL with a mixture of MeOH:water (50:50, v/v), and filtration by nylon syringe filters (13 mm, 0.22 µm) before injection in UHPLC-MS/MS.

### Egg extraction

**Fig. 2:**
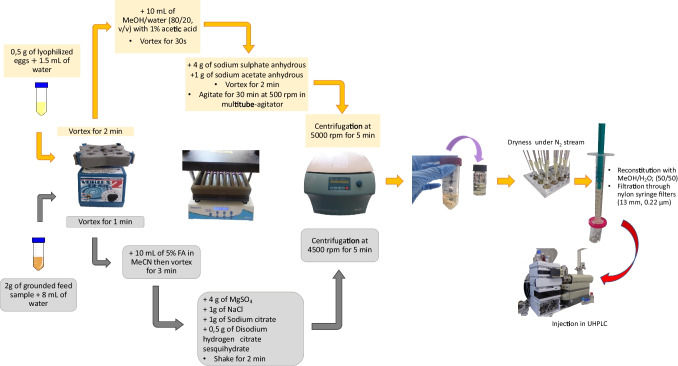
Representative schema of extraction procedure for feed and eggs

Egg samples were extracted according to the procedure described by Garrido-Frenich et al. (2011), with minor modifications. The total number of eggs used to produce the 35 final samples was 350 eggs (i.e., 10 eggs were homogenized and then lyophilized to produce 1 sample). First, 1.5 mL of water was added to 0.5 g of lyophilized eggs into a 50-mL centrifuge tube following Lehotay’s (2022) proposed equivalence between fresh and lyophilized eggs, and subsequently vortexed for 2 min. After, 10 mL of a MeOH/water solution (80/20, v/v) with 1% acetic acid was added and vortexed again for 30 s. Then, 4 g of sodium sulfate anhydrous and 1 g of sodium acetate anhydrous were added, and the mixture was vortexed for 2 min, then transferred into a multitube-agitator and agitated for 30 min at 500 rpm. After centrifugation at 5000 rpm for 5 min, 2 mL of the supernatant layer was transferred to a 4-mL glass tube, then evaporated under a gentle nitrogen flow at 45 °C, reconstituted in a mixture of MeOH:water (50:50, v/v), and filtrated with nylon syringe filters (13 mm, 0.22 µm) before injection in the UHPLC-MS/MS system. Both procedures are graphically represented in Fig. 2.

## Results

### Method evaluation

The performance of the method was evaluated in terms of linearity, matrix effect (ME), extraction recovery (RE), precision, limit of detection (LOD), and limit of quantification (LOQ). The results are shown in Table 2. It is worth noticing that the egg-related values shown in Table 2 are those of lyophilized (freeze-dried) eggs.

**Table 2:** Performance characteristics of the method

	**RE**^**a**^** (%)**		**ME**^**a**^** (%)**		**LOQ (µg/kg)**		**LOD (µg/kg)**	
**Analytes**	**Feed**	**Eggs**	**Feed**	**Eggs**	**Feed**	**Eggs**	**Feed**	**Eggs**
ENN B_1_	100.9	102.5	-19.6	-69.6	1.1	0.6	0.3	0.2
	**RSD (%) (n = 6)**							
	1.4	8.2	3.7	11.3				
ENN B	102.7	107.5	-24.2	-72.0	0.4	0.44	0.1	0.1
	**RSD (%) (n = 6)**							
	2.0	6.8	13.0	9.1				
BEA	87.6	106.4	-47.1	-74.2	0.8	0.3	0.2	0.1
	**RSD (%) (n = 6)**							
	3.2	5.4	3.7	8.6				
ENN A_1_	96.9	91.8	-23.0	-75.0	0.67	0.3	0.2	0.1
	**RSD (%) (n = 6)**							
	2.6	5.0	10.6	9.2				
ENN A	97.3	101.0	-31.4	-86.3	1.0	0.8	0.3	0.2
	**RSD (%) (n = 6)**							
	1.5	14.9	9.8	4.0				

^a^Studied concentration, 20 µg/kg

The linearity of the method was evaluated by spiking extract blank samples at six different concentration levels (i.e., 1, 2, 5, 10, 15, and 30 µg/L), and analyzing them in duplicate (instrumental replicates). All calibration curves showed good linearity, with coefficients of determination (*R*^2^) higher than 0.99. The determination of the LODs and LOQs was carried out considering the concentration of analyte giving a signal to noise ratio (S/N) of 3 and 10, respectively (Commission Regulation 2006; Mahdjoubi et al. 2020).

ME can be estimated by comparing the analytical response provided by blank extracts spiked after the sample treatment with the response that results from a standard solution at the same concentration. RE was estimated by comparing the peak area of samples spiked before and after the sample treatment to evaluate the analyte losses. MEs and REs were evaluated at 20 µg/kg by three experimental replicates injected twice (*n* = 6). The calculation of ME and RE was established using the following equations:

1 $$\mathrm{ME }(\mathrm{\%})=\frac{\left(\mathrm{signal\;of\;spiked\;extract}-\mathrm{signal\;of\;standard\;solution}\right)}{\mathrm{signal\;of\;standard\;solution}}\times 100$$

2 $$\mathrm{RE }(\mathrm{\%})=\frac{(\mathrm{signal\;of\;blank\;sample\;spiked\;before\;treatment})}{\mathrm{signal\;of\;spiked\;extract}}\times 100$$

Matrix effects were significant, particularly in egg samples, although quite constant as shown by the relative standard deviation. This means that calibration with standard solution is not possible and matrix-matched calibration is required. Procedural calibration might not be necessary as recoveries were satisfactory for both feed and eggs.

### Mycotoxin occurrence data

Ten samples of poultry feed and 35 samples of pooled lyophilized eggs, containing 10 eggs in each sample, were tested for ENNs and BEA. It is worth noticing that the usage of mycotoxin binder in feed is a very common practice among Algerian farmers. The results showed that only ENN B_1_ was found in 9 of 10 feed samples, with contamination levels ranging from 3.6 to 41.5 µg/kg, while BEA was detected only in one sample with a concentration of 12 µg/kg. However, none of the investigated mycotoxins was detected in the eggs. Results are shown in Table 3.

**Table 3:** ENN B_1_ and BEA occurrence in positive chicken feed samples

**ENN B**_**1**_		
Sample n^o^	Concentration (µg/kg)	RSD (%) (*n* = 4)
02	15.1	12.2
03	24.8	6.8
04	41.5	6.2
05	< LOQ	11.4
06	10.5	11.2
07	6.7	12.5
08	3.9	6.2
09	34.9	14.3
10	3.6	1.1
**BEA**		
08	0.4	8.5

## Discussion

The obtained results indicate that variations in mycotoxin contamination levels among the poultry feed samples lead to heterogeneity in mycotoxin occurrences in these samples. This observation resonates with the complex and dynamic nature of mycotoxin contamination, which is influenced by an array of factors including feed composition, storage conditions, and processing methods. The variability in feed ingredients, their sourcing, and preparation techniques can all contribute to the divergence in mycotoxin concentrations encountered. This emphasizes the need for a comprehensive understanding of the entirety of the feed formulation process. For this purpose, a sampling procedure was accomplished for both chicken eggs and feed. The collected samples were then analyzed using the procedure detailed in the above sections.

The results of the analysis have unveiled feed sample numbers 3, 4, and 9 as having the highest ENN B_1_ contamination levels, with concentrations of 24.8, 41.5, and 34.9 µg/kg respectively. These samples originate from distinct sources, representing three different farms situated in three selected regions. Sample 3 emerges from Guelma, sample 4 from Biskra, and sample 9 from Batna. Interestingly, these samples share a common feature of elevated maize concentrations within their feed compositions, comprising more than 57% of the total composition on average. Additionally, feed formulations consistently include an average of 20% soybean content. The remaining components encompass bran, barley, wheat, and limestone, along with an average of 3% of mineral vitamin supplement (MVS) and phosphate. Hence, feed formulation exhibits varying degrees of diversity across the samples, reflecting its complexities.

This intricate interplay between feed components is instrumental in understanding the mycotoxin transfer dynamics in the poultry production cycle. The presence of high levels of maize, a frequently used energy source in poultry feed, is particularly interesting. Previous studies have highlighted maize as a prominent matrix for mycotoxin occurrence, often being heavily contaminated (González-Jartín et al. 2021). This aligns with the findings that maize and animal compound feed are prevalent sources of emerging mycotoxins (Fumagalli et al. 2021). Consequently, the maize-rich feed compositions in samples 3, 4, and 9 could be an essential contributor to the elevated ENN B_1_ levels detected in these samples.

The existence of ENN B_1_ in these three samples may also be attributed to factors such as elevated temperatures in Biskra and high humidity levels in Guelma and Batna. These conditions, especially when combined with inadequate storage methods, can expedite the proliferation of *Fusarium* fungi on crops. Such circumstances can significantly impact mycotoxin generation and subsequent contamination, underscoring the potential implications of these occurrences.

The presence of emerging *Fusarium* mycotoxins like enniatins (ENNs) in food and feed has raised toxicity concerns. While in vitro studies suggest ENNs are toxic, most in vivo data indicate low or no toxicity. Interestingly, despite frequent ENN detection in poultry feed, a comprehensive analysis revealed an absence of detectable mycotoxin levels in eggs, warranting further investigation into the underlying mechanisms governing their transfer and bioavailability throughout the poultry production cycle (Gruber-Dorninger et al. 2017).

Rodríguez-Carrasco et al. (2020) research on enniatins indicates that these substances are efficiently broken down in the body of the laying hen due to their rapid phase 1 metabolism which involves the enzymatic modification of compounds within an organism resulting in chemical transformations that often make them more water soluble and thus facilitate their elimination from the body. Accordingly, this fast transformation may be partly responsible for the reduced amounts of parental enniatin molecules in the analyzed egg samples, since they may be digested before reaching detectable levels.

The research conducted by Tangni et al. (2020) sheds light on a critical aspect of mycotoxin transfer from feed to eggs within the context of poultry production. The findings highlight that the transfer rate from feed to eggs is indeed quite low. Specifically, after a period of 2–3 days of consuming contaminated feed, the transfer rates of ENN B, ENN B_1_, and BEA to eggs were measured at 0.1%, 0.05%, and 0.44% respectively. It is noteworthy that constant levels of contamination persisted for around 5–6 days, followed by the attainment of toxin-free eggs after 9–10 days when the laying hens were fed uncontaminated feed. This indicates a minimal and gradual transfer of these specific mycotoxins from feed to various poultry products, including eggs. While the observed low transfer rates suggest that these mycotoxins contribute only marginally to the overall dietary intake of consumers, the long-term implications cannot be disregarded. Over an extended duration, the continued consumption of feed with even minor levels of contamination could potentially lead to the accumulation of mycotoxins in chicken offal, meat products, and eggs. The prospect of such prolonged exposure raises concerns about mycotoxicosis, a condition that may manifest as chronic health effects due to continuous ingestion of mycotoxin-contaminated products. This aligns with the broader scientific concern emphasized by Lee and Ryu (2015) that the consumption of mycotoxin-contaminated foods carries not only the risk of chronic mycotoxicosis but also acute poisoning with potentially fatal consequences.

The presence of mycotoxin binders within the feed could also be a pivotal factor influencing the observed phenomenon. These binders, commonly used in feed formulations, have the capacity to adsorb mycotoxins within the gastrointestinal tract, reducing their bioavailability for absorption. Additionally, they might reduce contamination levels by changing the chemical composition of mycotoxins (Sarandan et al. 2012; Wang et al. 2022). Consequently, the presence of mycotoxin binders in the feed could potentially hinder the uptake of mycotoxins by the laying hens, thus influencing their absence in eggs. This underscores the intricate interplay between feed additives and mycotoxin dynamics within the poultry production process. According to Chowdhury and Smith (2004), laying hens are vulnerable to long-term exposure to *Fusarium* mycotoxin mixtures, but many of the negative effects can be avoided by supplementing their feed with mycotoxin binders. Jiang et al. (2014) have shown that these negative effects can be partially mitigated by the dietary addition of mycotoxin adsorbents at a rate of 2 g/kg to diets.

The consequences of mycotoxin contamination go beyond animal feed and present an entirely novel perspective in the evolution of food safety. Inadvertently introducing pollutants into the human food chain, contaminated feed can cause the release of dangerous substances and the production of metabolites in tissues, eggs, and derivatives. Although emergent mycotoxins are generally thought to be less harmful than their regulated counterparts, interactions between the two mycotoxin groups have the potential to increase their combined toxicity (Rossi et al. 2020). When regulated and emerging mycotoxins coexist on natural matrices, more damage is observed (Pérez-Fuentes et al. 2021). It is hypothesized that BEA and ENNs have a primary toxic effect due to their ionophoric characteristics, which allow them to form stable and lipophilic complexes with cations, and transport them to lipophilic matrices like cell membranes, causing osmotic balance disruptions. Furthermore, ENNs and BEA generate cation-selective channels in cell membranes, impairing membrane activities (Kamyar et al. 2004). Thus, emerging mycotoxins cause mitochondria-mediated cytotoxicity in human neuroblastoma cells, suggesting that they may be detrimental to human health if left unchecked (Chiminelli et al. 2022).

According to Liu et al. (2021), the results from the analysis of five mycotoxins (BEA, ENN A, ENN A_1_, ENN B, and ENN B_1_) in 114 egg samples, 45 commercial eggs, and 69 rural eggs revealed that BEA had a detection rate of 30.4% in rural egg samples with a concentration range of 0.3–9.8 μg/kg. In commercial egg samples, only one sample was contaminated with BEA at a concentration of 4.3 μg/kg. However, the four ENNs were found at concentration levels below the limit of quantitation: 0.5, 0.2, 0.3, 0.5, and 0.3 μg/kg for BEA, ENN A, ENN A_1_, ENN B, and ENN B_1_, respectively. These results are in line with the findings of the current work.

In the study carried out by Jestoi et al. (2009), in which BEA and ENN were discovered in egg samples for the first time, analyses of the egg samples (i.e., 112 whole eggs and 367 egg yolks) showed that BEA and ENN B and ENN B_1_ can be present in eggs. However, most of the mycotoxins were found at trace levels, specifically concentrations lower than the limit of quantification: 1.0, 0.03, 0.42, 0.40, and 1.12 μg/kg for BEA, ENN A, ENN A_1_, ENN B, and ENN B_1_, respectively. On the other hand, ENN A and ENN A_1_ were not found in any of the whole egg samples, which is also in agreement with the results obtained in the present investigation.

The present study highlights the presence of ENN B_1_ and BEA in poultry feed, with varying contamination levels, while their absence in eggs underscores the complexity of mycotoxin transfer within the poultry production system. These findings emphasize the need for ongoing research and vigilant monitoring to comprehensively address mycotoxin risks and ensure the safety of the food supply chain.

## Conclusions

In conclusion, the results obtained in the present study showed a low level of mycotoxin contamination in the analyzed chicken feed samples, and no contamination in egg samples. Moreover, it is worth noting that mycotoxin binders mixed with animal feed may result in low concentrations of emerging mycotoxins; this effect can be seen in the feed and eggs investigated in this study.

The quantities of emerging mycotoxins in animal products may be modest. However, long-term ingestion of those quantities may increase people’s dietary exposure to mycotoxins and put their health at risk. Thus, further research is required to identify the critical metabolites of BEA/ENNs. Therefore, controlling the levels of BEA and ENNs in the feed is crucial, mainly due to the lack of regulation regarding limits of emerging mycotoxins in feed and food in different countries.

## Data Availability

The authors declare that the data supporting the findings of this study are available within the paper. Should any raw data files be needed in another format, they are available from the corresponding author upon reasonable request.
